# Seroprevalence and risk factors of tropical theileriosis in smallholder asymptomatic large ruminants in Egypt

**DOI:** 10.3389/fvets.2022.1004378

**Published:** 2022-10-11

**Authors:** Hend M. El Damaty, Sarah G. Yousef, Farouk A. El-Balkemy, Omid Nekouei, Yasser S. Mahmmod, Ibrahim Elsohaby

**Affiliations:** ^1^Infectious Diseases, Department of Animal Medicine, Faculty of Veterinary Medicine, Zagazig University, Zagazig, Egypt; ^2^Department of Infectious Diseases and Public Health, Jockey Club College of Veterinary Medicine and Life Sciences, City University of Hong Kong, Hong Kong, Hong Kong SAR, China; ^3^The Centre for Applied One Health Research and Policy Advice (OHRP), City University of Hong Kong, Hong Kong, Hong Kong SAR, China; ^4^Department of Veterinary Sciences, Faculty of Health Sciences, Higher Colleges of Technology, Abu Dhabi, United Arab Emirates

**Keywords:** *Theileria annulata*, risk factor, asymptomatic carrier, large ruminant, seroprevalence, smallholder-production system

## Abstract

Knowledge of the prevalence and epidemiological determinants of tropical theileriosis in large ruminants, particularly in the asymptomatic carrier, is crucial for designing and implementing effective host-specific control measures. This study aimed to estimate the seroprevalence of tropical theileriosis in asymptomatic cattle and water buffaloes and identify the potential risk factors of theileriosis in large ruminants raised under smallholder-production system in Egypt. A cross-sectional study was conducted in five districts of the Sharkia governorate from March 2019 to February 2020. In total, 350 serum samples were collected from cattle and water buffaloes under smallholder-production system and tested for *Theileria annulata* antibodies using the indirect antibody fluorescence test (IFAT). Data on species, host characteristics, presence of ticks, season, and districts were collected at sampling using a questionnaire. A multivariable mixed-effects logistic regression model was built to determine the potential risk factors associated with *T. annulate* seropositivity of the animals. The overall apparent seroprevalence of *T. annulata* in 350 tested animals was 70%. In the univariable analyses, cattle compared to buffaloes, younger animals compared to older ones, animals with ticks on their bodies, and warmer seasons were all associated with a higher likelihood of seropositive results in the study population while sex of the animals was not associated with seropositivity. The final multivariable model showed that animals with ticks on their bodies had 3.5× higher odds of seropositivity than those with no ticks (*P* < 0.001), and warmer seasons were associated with the higher odds of infection compared to winter (*P* = 0.003). The high seroprevalence of tropical theileriosis in the study region indicates that the disease is endemic among smallholders of large ruminants. The identified risk factors of *T. annulata*-seropositivity in asymptomatic carrier animals provides evidence-based guidance for adopting effective intervention measures.

## Introduction

Tropical theileriosis is a protozoan disease transmitted by members of the Ixodidae family of ticks and causes significant economic losses in livestock production in tropical and subtropical regions (1–3). The disease threatens about 250 million cattle, substantially impacting livestock production in many developing countries (4). Tropical theileriosis in large ruminants is a highly debilitating disease caused by *Theileria annulata*, which is distributed according to the natural habitat of its tick vector (5). *T. annulata* has a complex life cycle through which it is transmitted transstadially by *Hyalomma* spp. ticks (6) and the most common tick species infected with *T. annulata* in Egypt is *Hyalomma excavatum* (7). Approximately, 80% of the global cattle population is exposed to tick infestation (8), causing an estimated loss of 13.9 to 18.7 billion US dollars and an annual loss of 3 billion pieces of hide in cattle (9, 10). The mortality rates of *T. annulata* are much higher in imported breeds than in native ones (11). *Theileria* infection can reduce milk production in cattle by 2.76 L/day/cow (12). Furthermore, the native cattle that have been chronically infected and recoverd are long-term carriers of *T. annulata* because only a few of their erythrocytes are infected with the parasite (13). These asymptomatic carriers play an important role in the cycle of infection, as reservoirs for tick infection and the spread of theileriosis between large ruminants (14).

Diagnosis of theileriosis is based on clinical observations and microscopic examination of Giemsa-stained blood and lymph node smears in acute cases, which incurs technical difficulties and has a low sensitivity in detecting the asymptomatic carriers (15, 16). Thus, serological tests continue to be used as the most cost-efficient methods in large-scale studies to identify carrier animals and assess the distribution of infection (17, 18). Currently, there are many serological tests available for this purpose, but the indirect antibody fluorescence test (IFAT) remains the most economical and reliable test in epidemiological studies (3), despite some limitations, such as cross reactivity with other *Theileria* spp. as well as the subjective and operator-dependent interpretation of the fluorescent results (19, 20). Molecular diagnostic tools can also be used to detect very low levels of parasitemia and differentiate between different *Theileria* spp. (14). However, it is not used commonly for large-scale surveys because it is costly and requires specialized technical skills (21).

In Egypt, there are 8.6 million cattle and buffaloes, 80% of them are owned by smallholder farmers (22). The detection and prevalence of *T. annulata* in cattle have been documented in other countries (23–25), including Egypt (26–30). However, there is little information about the detection and epidemiology of theileriosis in water buffaloes. Only few studies have reported the prevalence of theileriosis in buffaloes in India (31) and Egypt (15). However, no studies investigated the potential role of asymptomatic carriers in the spread of the disease. An investigation of the differences in theileriosis prevalence among cattle and buffalo hosts in smallholders is crucial for implementing effective host-specific control measures. Although smallholder producers constitute the most common livestock farming in Egypt (32), no studies have been published examining the epidemiological determinants of theileriosis in bovines raised under the smallholder-production system (33). The objectives of this study were to estimate the seroprevalence of bovine theileriosis in cattle and buffaloes, and to identify the potential risk factors associated with theileriosis in large ruminants raised under the smallholder-production system in Sharkia governorate, Egypt.

## Materials and methods

### Study area

**Figure 1:**
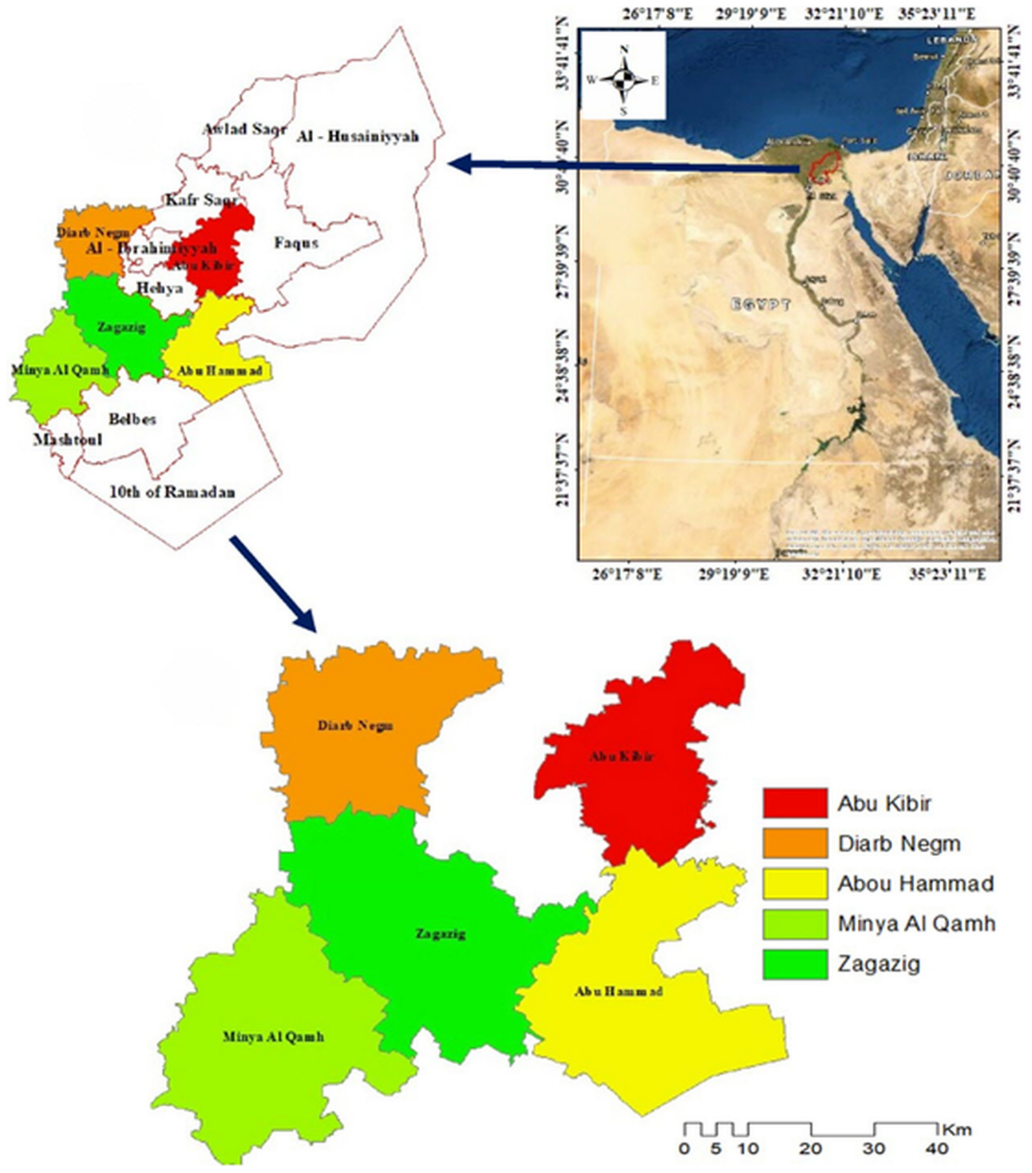
Map of Egypt showing the location of Sharkia governorate and the five districts included in the study. The map was created using ArcGIS v10.4.

The study was conducted in five districts of the Sharkia governorate between March 2019 and February 2020. Sharkia governorate is one of the largest agricultural governorates in Egypt, located in the Eastern Nile Delta (Figure 1). Sharkia governorate is Egypt's third most populous governorate, with a high density of ruminants (cattle, sheep and goats) which are mainly raised for meat production. Sharkia governorate was chosen due to a complete lack of data on theileriosis compared to the other governorates in Egypt. Furthermore, Sharkia governorate has rural villages that primarily rely on raising livestock under smallholder-production system.

### Study design

A cross-sectional study was designed to sample cattle and buffalo from smallholder farms in five districts of the Sharkia governorate. Smallholder farms were defined as those holding <10 cattle and buffaloes and selected based on their location and animal density. A two-stage random sampling approach was used. In the first stage, each district was considered one stratum and smallholder farms within each district were randomly selected (Table 1). In the second stage, cattle and buffaloes were randomly selected from each selected smallholder farm. The sample size required was calculated assuming a 50% expected seroprevalence, a 95% confidence level, and a 5% absolute precision using the following formula (34):


$$\begin{array}{l} n=\frac{1.96^{2}\;P_{exp\;}\;\left(1-\;P_{exp}\right)}{d^{2}} \end{array}$$

Where *n* = required sample size, *P*_*exp*_ = expected prevalence and *d* = desired precision.

**Table 1:** Frequency distribution of smallholder farms and large ruminants selected for the study and tested for *T. annulata-*antibodies using IFAT from five districts of the Sharkia governorate, Egypt.

**Districts**	**No. of smallholder farms**	**Cattle**		**Buffalo**		**Total number of** **infected animals (%)**
		**No**.	**Infected (%)**	**No**.	**Infected (%)**	
Abou Hammad	10	30	21 (70.0)	10	7 (70.0)	28 (70.0)
Abu Kibir	14	36	28 (77.8)	16	10 (62.5)	38 (73.1)
Diarb Negm	20	50	29 (58.0)	22	10 (45.5)	39 (54.2)
Minya Al Qamh	22	71	56 (78.9)	23	14 (60.9)	70 (74.5)
Zagazig	25	77	61 (79.2)	15	9 (60.0)	70 (76.1)
Total	91	264	195 (73.9)	86	50 (58.1)	245 (70.0)

The total number of cattle and buffaloes enrolled in the study was justified by the expected prevalence of theileriosis (15) and the distribution of cattle and buffaloes in each district of the Sharkia governorate (Table 1).

In total, 350 animals (264 cattle and 86 buffaloes) were randomly selected from the 91 smallholder farms. Each selected animal was thoroughly examined, and a complete case record was obtained according to the method described by Constable, Hinchcliff (35). Animals with clinical signs of theileriosis, such as enlarged lymph nodes, fever, nasal discharge, and corneal opacity, were excluded from the study. Animals with a recent history of anthelmintic administration at the time of sampling were also excluded.

### Sample and data collection

Blood samples were collected from the jugular veins of selected cattle and water buffaloes by venipuncture, using a 20-gauge, 1-inch hypodermic needle into a sterile, plastic Vacutainer tube without anticoagulant. Each sample was labeled with a unique animal and smallholder identifier number. Samples were then transported in a cold box to the laboratory at the Department of Animal Medicine, Zagazig University, Egypt. Sera were extracted by centrifugation of the blood samples at 1,500 × g for 10 min at 20°C within 5 h of collection. Sera were stored in – 20°C freezer for further analysis with IFAT to detect the specific antibodies against *T. annulata*. Epidemiological data, including sex, age, season, animal species, and the presence of ticks on animals were collected using a questionnaire at sampling. Sampling was carried out after obtaining an informed consent from the animal owners.

### Indirect fluorescent antibody test

The IFAT was performed as described by Burridge and Kimber (36), using *T. annulata* piroplasm antigen-coated slides and standard positive and negative control sera, which were kindly provided by the Veterinary Serum and Vaccine Research Institute, Egypt. Anti-bovine immunoglobulin was prepared in rabbit using the method described by Abd Elwanis and Khodeir (37) and labeled with fluorescein isothiocyanate. This conjugate was diluted 1:80 and was reacted specifically with bovine IgG. The antigen slides were incubated at – 70°C for 30 min; fingernail polish circles were applied over the slide and air-dried. All sera were inactivated at 56°C for 30 min. The diluted sera (1:20) were used (5–10 μL/well), and standard positive and negative control sera were added on each slide. The slides were then incubated in a moist chamber for 30 min at room temperature. The slides were then washed twice with phosphate-buffered saline (PBS). Approximately, 10 μL of the diluted conjugate (1:80) was added to each well and incubated for 30 min before washing and mounting with PBS/glycerol in equal amounts. The fluorescence on slides was examined under ultraviolet light using a Carl Zeus Jenny fluorescent microscope at ×40 magnification. The appearance of a yellow-green fluorescence color was considered a positive reaction (38).

### Statistical analysis

The serological status of each sample/animal was defined as the dichotomous outcome of interest (positivie or negative antibodies against *T. annulata*). The explanatory variables (potential risk factors) available in the study were animal species (cattle/buffalo), sex (male/female), age (≤2 years and >2 years), presence of ticks (yes/no), location (the five study districts), and season (summer, autumn, winter, and spring).

All data analyses were carried out in Stata v17 (Stata Corp, College Station, TX). The frequency distributions of tested animals by the potential factors of interest were assessed and tabulated. Univariable and multivariable mixed-effects logistic regression models were built to investigate the association between *T. annulata* serum status and the risk factors of interest in the study region, including the geographical locations as the random effect. First, univariable/unconditional associations between the outcome and each explanatory variable were evaluated. Variables with a conservative *P*-value ≤0.20 (39) were retained for the multivariable modeling process. A backward elimination strategy was used in building the final multivariable mixed-effects logistic model. Variables with *P* < 0.05 were included in the final model. Two-way interactions between variables in the final model were also assessed.

## Results

In total, 264 cattle and 86 buffalos were tested for *T. annulata* antibodies in this study, with a seroprevalence of 74 and 58%, respectively. The numbers of smallholder farms and animals selected and tested from each district of the Sharkia governorate are presented in Table 1. There was not a substantial difference in the animal-level seroprevalences among the studied districts (70–76%), except for a lower seroprevalence from Diarb Negm district (54%). The frequency distributions of the study population by the variables of interest as well as the results of univarbale mixed-effects logistic models are summarized in Table 2. The overall apparent seroprevalence of *T. annulata* in 350 tested animals was 70%. In the univariable analyses, all explanatory variables of interest showed a significant association with the seroprevalence of *T. annulata* in the study population, except for ‘sex' (*P* = 0.237) that did not proceed to the multivariable modeling process (Table 2). Cattle compared to buffaloes, younger animals compared to older ones, animals with ticks on their bodies, and warmer seasons were all associated with higher odds of seropositive results in the study population (Table 2).

**Table 2:** Frequency distribution and the ouput of univariable mixed-effects logistic regression models evaluating the association between each risk factor of interest and seropositivity to *T. annulata* in 350 large ruminants raised under the smallholder–production system in five districts of the Sharkia governorate, Egypt.

**Variable**	**No. of seropositive**	**No. of seronegative**	**OR** **(95% CI)^1^**	***P*-value^2^**
**Species**				
Buffalo	50	36	–^3^	
Cattle	195	69	1.98 (1.18–3.32)	0.009
**Sex**				
Male	81	28	–	
Female	164	77	0.66 (0.39–1.12)	0.124
**Age**				
Young ≤2 years	134	42	–	
Old >2 years	111	63	0.44 (0.27–0.74)	0.002
**Presence of ticks**				
Absent	112	79	–	
Present	133	26	3.86 (2.28–6.51)	0.000
**Season**				0.0001^4^
Winter	16	21	–	
Spring	32	6	6.91 (2.31–20.71)	0.003
Summer	138	66	2.71 (1.31–5.60)	0.007
Autumn	59	12	6.29 (2.46–16.07)	0.000
**Total**	245	105		

1 OR, odds ratio; CI, confidence interval.

2 *P*-value ≤ 0.2 was used for screening the variables in univariable analyses.

3 Reference/baseline categories.

4 Overall *P*-value for season effect.

The final multivariable model revealed that the presence of ticks on animals and season were significantly associated with seropositivity to *T. annulata* in the studied population (Table 3). Animals with ticks on their bodies had 3.5× higher odds of seropositivity than those with no ticks (*P* < 0.001). In general, warmer seasons were associated with higher odds of infection compared to winter (*P* = 0.003). Location accounted for ~1.5% of the total variation in the model (*P* = 0.198), suggesting the uniformity in the distribution of infection among the districts in the region.

**Table 3:** Output of the final multivariable mixed-effects logistic regression model indicating variables associated with *T. anaulata*-seropositivity of cattle and buffaloes raised under the smallholder–production system in five districts of the Sharkia governorate, Egypt.

**Variable**	**No. of animals**	**OR (95% CI)^1^**	***P*-value^2^**
**Presence of ticks**			
Absent	191	–^3^	
Present	159	3.5 (2.05–5.98)	0.000
**Season**			0.003
Winter	37	–	
Spring	38	6.1 (1.94–18.98)	0.002
Summer	204	2.3 (1.05–4.97)	0.038
Autumn	71	4.8 (1.75–12.93)	0.002
**Random effect**	**Estimate**		
District-level variance	0.049	(0.002–1.19)	0.198

1 OR, odds ratio; CI, confidence interval.

2 *P*-value < 0.05 was considered statistically significant.

3 Reference/baseline categories.

## Discussion

Tropical theileriosis is a widespread tick-borne disease that affects large ruminants in Egypt, resulting in major losses in meat, milk, and leather production as well as animal death (40). In the present study, *T. annulata* antibodies were detected in sera samples collected from cattle and buffaloes in five districts of the Sharkia governorate, Egypt using IFAT. The potential risk factors associated with *T. annulata* seropositivity were also identified.

In the present study, the apparent seroprevalence of *T. annulata* in cattle was 74%, which is higher than the 20.89% (16) and 33.33% (41) reported previously in cattle and water buffaloes in the Delta region of Egypt, the 34.9% reported in Eastern Turkey (23) and the 31% reported in Sudan (42). Furthermore, the high seroprevalence reported in cattle in this study was comparable to the 68% found in Sudan (43) and the 67.5% reported in the Cappadocia region of Turkey (44). However, it was lower than the seroprevalence (77.9%) reported in cattle in the Kurdistan region of Iraq by indirect enzyme-linked immunosorbent assay (45). The apparent seroprevalence of *T. annulata* in buffaloes was 58% in this study, which is higher than the 53.3% reported by PCR in buffaloes in Lahore district of India (31) but lower than the 88% previously reported in Egyptian buffaloes (15).

The high seropositivity observed in this study can partially be attributed to the rural environment in which cattle and buffaloes were raised as part of the smallholder-production system in the Sharkia governorate. This high seroprevalence could be explained by a number of factors, including the lack of effective tick control programs and farmer's low education levels, which together may result in inefficient animal management in this type of breeding system. Furthermore, climatic changes occurring over recent decades have increased the number and spread of vector ticks (46). Nonetheless, seroprevalence should be interpreted with caution due to differences in diagnostic tests sensitivity, the number of tested serum samples, differences in management practices between locations, and variations in environmental and climatic conditions, which are mainly associated with the tick distribution (47, 48).

In the univerailble analysis, *T. annulata* seropositivity was significantly higher in cattle than in water buffaloes. This finding is consistent with Fadly (49), who found that cattle have a higher seroprevalence to *T. annulata* than buffaloes in the El-Behera governorate, Egypt. This could be explained by the fact that water buffaloes have thicker skin and lower sensitivity to tick proteins compared to cattle and therefore less susceptible to tick infestation (15, 50, 51). In contrast, a recent study reported no significant differences in *T. annulata* seropositivity between cattle and buffaloes in India (52).

The univariable analysis in this study showed that the odds of *T. annulata* seropositivity were lower in older animals (>2 years old) than in young animals (≤2 years old). Similarly, several studies have reported a high prevalence of theileriosis in young cattle (43, 53, 54). However, Abaker, Salih (25) found that calves aged <1 year in Sudan had the lowest prevalence, while older animals (>3 years) had the highest prevalence. Furthermore, an Indian study reported that theileriosis infection rates were higher in older animals, whereas no infection was recorded in calves (52). According to Salih, El Hussein (24), the animal age is not a risk factor for infection; however, the study reported higher prevelance of *T. annulata* infection in animals aged >4 years, which attributed to the cumulative *Theileria* infection associated with increased protective immunity against clinical infection. Our findings also revealed that animal sex is not significantly associated with *T. annulata* seropositivity. This finding could be attributed to the similar management practices that were followed for all animals regardless of their sex (26, 55). Also, a recent study in Egypt reported no significant association between animal sex and *Theileria* infection (56). However, another study in China reported higher risk of infection in male cattle than females, but did not provide a clear explanation for this finding (57).

The presence of ticks on animals increased the risk of *T. annulata* seropositivity in our study. In agreement with this finding, Kispotta, Islam (58) found that tick-infested cattle had three times risk of being infected than tick-free cattle. This finding is not surprising given ticks' role in transmitting various blood parasites, including *T. annulata*. However, in a study conducted in Pakistan, Khattak, Rabib (59) reported no significant association between theileriosis infection and tick infestation, indicating a low level of parasitemia or low diagnostic test sensitivity.

The risk of *T. annulata* seropositivity in this study was associated with the warm seasons, with the highest seroprevalence recorded in spring (84.2%) and autumn (83.1%) followed by summer (67.7%). Similarly, a higher prevalence of theileriosis in autumn and spring than in winter has previously been reported in Dakahlia, El-Beheira, and Sharkia governorates, Egypt (28, 49, 60). The high risk of *T. annulata* seropositivity in warmer seasons can be attributed to the hot and humid weather during these months, leading to increases in tick activity (46, 61). In contrast, a study performed in Upper Egypt reported no significant difference in disease occurrence between hot and non-hot months (62). This could be due to the hot and dry climate in Upper Egypt all over the year, which is suitable for the activity dynamics of the ticks (63).

Although molecular tools are the most effective for detecting carriers of theileriosis, they are not cost-effective for large-scale surveys. When parasitemia levels are low, serological methods could be used for determining carrier status (64). In this study, IFAT was used to determine *T. annulata* seroprevalence. However, an important limitation of this study is the IFAT diagnostic accuracy (65) and cross-reactivity with other *Theileria* spp. However, to the author's knowledge, there is no published data on other *Theileria* spp. (except for *T. annulate)* in the study districts. Further studies on the distribution and epidemiology of *T. annulata* using molecular techniques in Egypt are recommended.

## Conclusions

The seroprevalence of *T. annulata* is high in asymptomatic large ruminants raised under the traditional smallholder-production system, indicating the endemicity of infection in the Sharika governorate, Egypt. Because there is a strong link between seropositivity and the presence of ticks on the animals, controlling ticks is critical in reducing the prevalence and spread of *T. annulata*. Thus, to reduce the risk of theileriosis and ensure a more sustainable control strategy, smallholder farmers' knowledge and awareness of the routes of theileriosis transmission must be improved, as well as encouraging farmers to adopt effective intervention measures, such as tick control and vaccination, particularly during the warm seasons.

## Data availability statement

The original contributions presented in the study are included in the article, further inquiries can be directed to the corresponding author/s.

## Ethics statement

The animal study was reviewed and approved by Institutional Animal Care and Use Committee (IACUC) of the Zagazig University (protocol no.: ZU-IACUC/2/F/25/2019). Written informed consent was obtained from the owners for the participation of their animals in this study.

## Author contributions

HE and FE-B contributed to the conception and design of the study. SY and HE carried out the field and laboratory work. YM, ON, and IE performed the statistical analyses and interpretation of the results. HE, SY, and YM wrote the manuscript's initial draft. ON and IE edited the initial manuscript. All authors contributed to the article and approved the submitted version.

## Conflict of interest

The authors declare that the research was conducted in the absence of any commercial or financial relationships that could be construed as a potential conflict of interest.

## Publisher's note

All claims expressed in this article are solely those of the authors and do not necessarily represent those of their affiliated organizations, or those of the publisher, the editors and the reviewers. Any product that may be evaluated in this article, or claim that may be made by its manufacturer, is not guaranteed or endorsed by the publisher.
